# Tregs with an MHC class II peptide–specific chimeric antigen receptor prevent autoimmune diabetes in mice

**DOI:** 10.1172/JCI168601

**Published:** 2023-09-15

**Authors:** Justin A. Spanier, Vivian Fung, Christine M. Wardell, Mohannad H. Alkhatib, Yixin Chen, Linnea A. Swanson, Alexander J. Dwyer, Matthew E. Weno, Nubia Silva, Jason S. Mitchell, Paul C. Orban, Majid Mojibian, C. Bruce Verchere, Brian T. Fife, Megan K. Levings

**Affiliations:** 1 Center for Immunology,; 2 Center for Autoimmune Disease Research, and; 3 Department of Medicine, Division of Rheumatic and Autoimmune Diseases, University of Minnesota Medical School, Minneapolis, Minnesota, USA.; 4 Department of Surgery and; 5 BC Children’s Hospital Research Institute, University of British Columbia, Vancouver, British Columbia, Canada.; 6 Department of Laboratory Medicine and Pathology, University of Minnesota Medical School, Minneapolis, Minnesota, USA.; 7 School of Biomedical Engineering, University of British Columbia, Vancouver, British Columbia, Canada.

**Keywords:** Autoimmunity, Immunology, Diabetes, Immunotherapy, Tolerance

## Abstract

Adoptive immunotherapy with Tregs is a promising approach for preventing or treating type 1 diabetes. Islet antigen–specific Tregs have more potent therapeutic effects than polyclonal cells, but their low frequency is a barrier for clinical application. To generate Tregs that recognize islet antigens, we engineered a chimeric antigen receptor (CAR) derived from a monoclonal antibody with specificity for the insulin B chain 10–23 peptide presented in the context of the IA^g7^ MHC class II allele present in NOD mice. Peptide specificity of the resulting InsB-g7 CAR was confirmed by tetramer staining and T cell proliferation in response to recombinant or islet-derived peptide. The InsB-g7 CAR redirected NOD Treg specificity such that insulin B 10–23–peptide stimulation enhanced suppressive function, measured via reduction of proliferation and IL-2 production by BDC2.5 T cells and CD80 and CD86 expression on dendritic cells. Cotransfer of InsB-g7 CAR Tregs prevented adoptive transfer diabetes by BDC2.5 T cells in immunodeficient NOD mice. In WT NOD mice, InsB-g7 CAR Tregs prevented spontaneous diabetes. These results show that engineering Treg specificity for islet antigens using a T cell receptor–like CAR is a promising therapeutic approach for the prevention of autoimmune diabetes.

## Graphical abstract

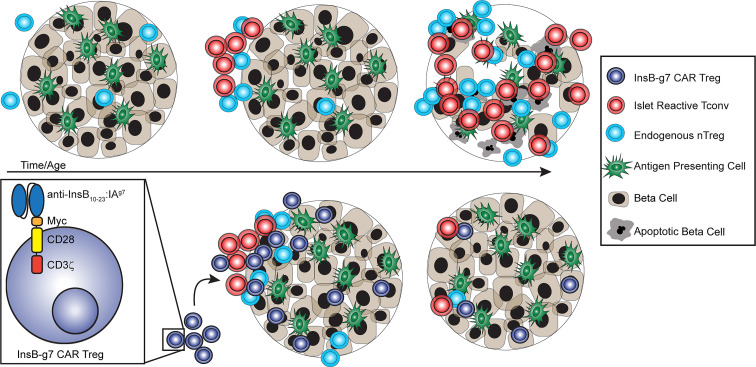

## Introduction

Autoimmune type 1 diabetes (T1D) is caused by T cell–mediated destruction of the insulin-producing β cells of the islets of Langerhans in the pancreas. Tregs, defined by expression of the *FOXP3* transcription factor, are suppressive CD4^+^ T cells that normally function to limit autoreactive effector T cell responses and prevent autoimmunity (1–3). Tregs from individuals with T1D have abnormal cytokine and gene expression profiles and reduced suppressive function, leading to the concept that strategies to restore Treg function could be a promising way to treat or prevent autoimmunity (4–8).

Substantial research using animal models of T1D has shown that therapeutic restoration of Tregs can prevent disease progression. Clinical studies have shown the safety of this approach in humans, but so far, evidence for efficacy is limited (9–11). A consideration is that, to date, all clinical trials of Treg therapy in T1D have used polyclonal cells, meaning that only a small fraction of the infused cells were specific for disease-relevant antigens (12). In animal models of autoimmune diabetes, there is clear evidence that islet antigen–specific Tregs are markedly more effective than polyclonal Tregs at preventing or delaying disease. In animal models of autoimmune diabetes, BDC2.5 TCR–transgenic Tregs, which are specific for a fusion peptide between insulin C and chromogranin A, or NOD T cells engineered to express FOXP3 and the BDC2.5 T cell receptor (TCR) suppressed diabetes induced by T cells from diabetic NOD mice or BDC2.5 T effector cells (13–16). Similarly, in WT NOD mice, a single infusion of BDC2.5 TCR–transgenic Tregs prevented and reversed spontaneous T1D, whereas polyclonal cells had no effect (15, 16).

In addition to engineering Tregs with TCRs, an alternate method to redirect specificity is through use of chimeric antigen receptors (CARs), engineered receptors in which antigen specificity is typically mediated by an extracellular single-chain antibody (scFv) and intracellular signaling driven by one or more costimulatory domains and CD3ζ. In comparison with using TCRs, using CARs to control T cell specificity has advantages that include the high affinity and specificity of scFvs, modular intracellular domain format, lack of mispairing with endogenous TCR chains, and MHC independence. We and others have shown that CAR Tregs specific for allo- or autoantigens have potent therapeutic effects in models of allograft rejection or autoimmunity (17–24). A limitation of CARs is that they are most effective when crosslinked by membrane or oligomeric proteins, possibly explaining why Tregs expressing a CAR specific for soluble insulin did not prevent or delay T1D in the NOD spontaneous diabetes model (25).

Seeking to design CARs for use in Tregs to treat T1D, we considered that the immunosuppressive function of Tregs needs to be present in both affected tissues and local LNs (26). We thus hypothesized that the ideal CAR Treg target in T1D would be a protein with expression restricted to islets and also present within the pancreas-draining LNs. Therefore, we developed a mAb specific for the aa residues 10 to 23 of the insulin B chain (InsB) presented in the context of NOD mouse MHC class II (MHCII), IA^g7^. We then converted this mAb to a CAR and tested its ability to redirect Treg specificity and create a therapeutic cell product that could suppress diabetogenic T cells and prevent autoimmune diabetes.

## Results

### Generation and validation of an InsB_10–23_:IA^g7^–specific mAb.

**Figure 1:**
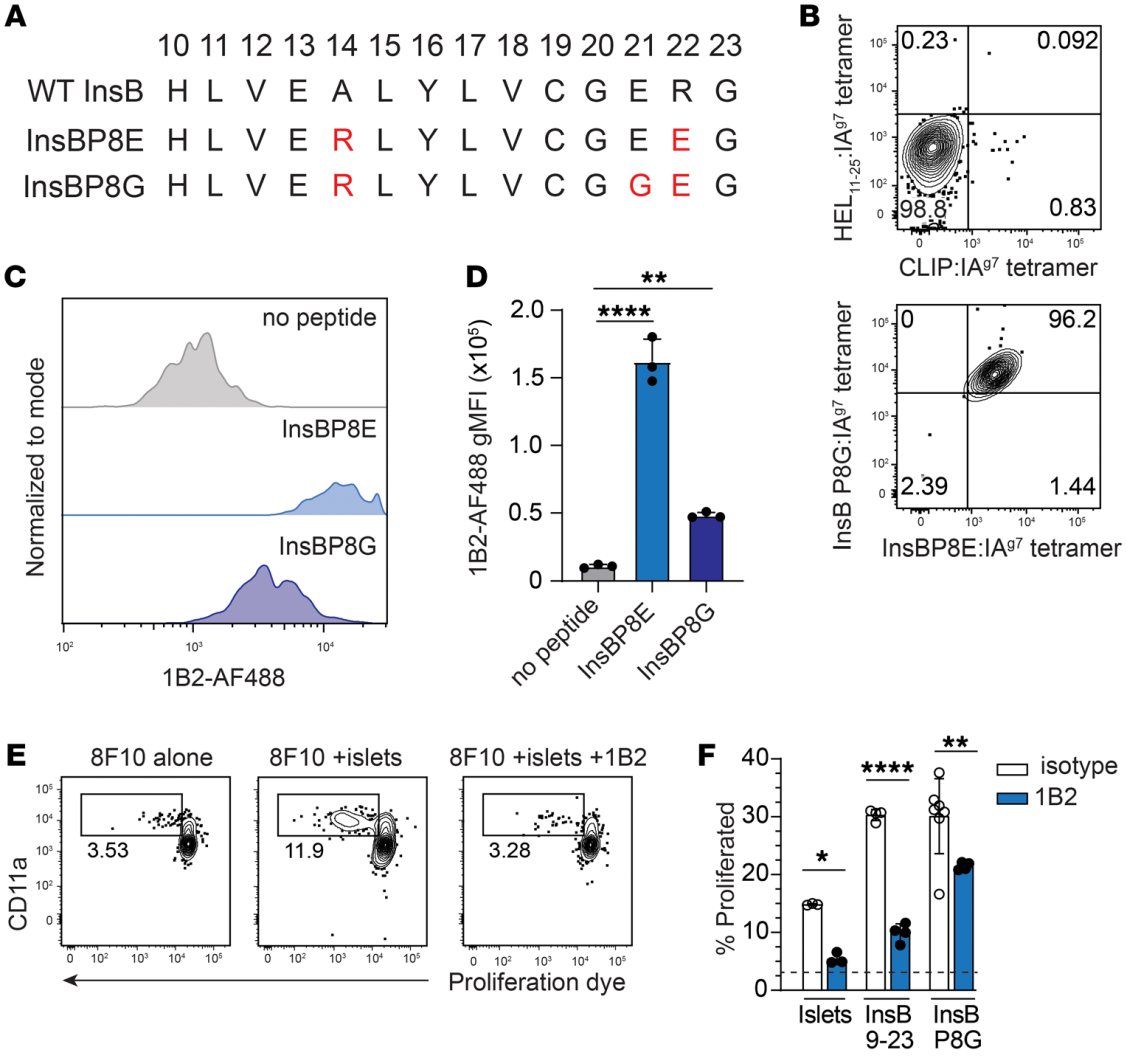
Generation and validation of an InsB_10-23_:IA^g7^–specific mAb. (**A**) An aa sequence of WT InsB_10–23_ compared with its mimotopes InsBP8E and InsBP8G. Position of each amino acid within the InsB peptide is shown on top. Residues highlighted in red font differ from the WT peptide sequence. (**B**) Flow cytometry plots showing tetramer staining of the 1B2 hybridoma. (**C**) Flow cytometry histograms showing AF488-labeled 1B2 antibody staining of BMDCs pulsed overnight with the indicated peptides. (**D**) Quantification of the data shown in **C**. Data are representative of 4 independent experiments. One-way ANOVA with multiple comparisons. **P < 0.01; ****P < 0.0001. (**E**) Representative flow cytometry plots of 8F10 TCR transgenic CD4^+^ T cells labeled with cell trace violet and stimulated in vitro for 4 days with APCs and islets in the presence or absence of 50 μg 1B2 blocking antibody. (**F**) Quantification of **E**, comparing the frequency of unstimulated 8F10 T cells with that of those stimulated with InsB10-23, InsBP8G, or NOD islets in the presence of 50 μg blocking 1B2 or IgG1 isotype control antibody. The dashed line represents the average proliferation of 8F10 T cells in the absence of antigen and mAbs. Data are pooled from 2 independent experiments. *n* = 3–9/group. One-way ANOVA with multiple comparisons. *P < 0.05; **P < 0.01; ****P < 0.0001.

Our goal was to design a CAR that enhanced retention of, and suppression by, Tregs at the site of islet antigen presentation. Therefore, we first generated mAbs with high specificity for an islet antigen peptide presented by NOD MHCII, IA^g7^. Specifically, we targeted InsB aa residues 10 to 23 because transgenic and retrogenic CD4^+^ T cells targeting this epitope are capable of causing autoimmune diabetes and CD4^+^ T cell responses to this epitope are required for spontaneous disease in NOD mice (27–30). To generate an InsB_10–23_:IA^g7^ mAb, B cell hybridomas were created from NOD mice immunized with recombinant IA^g7^ molecules containing InsB_10–23_ altered peptides InsBP8E and InsBP8G (31, 32) (Figure 1A). These modified insulin peptides were used because of their high affinity and well-defined binding to IA^g7^. In contrast, the native WT InsB_10–23_ binds weakly to IA^g7^, is unstable, and is presented in multiple registers (33–35). One of the resulting hybridomas, UMN-BF-1B2, hereafter referred to as 1B2, produced an IgG1 antibody that specifically bound to IA^g7^ tetramers containing InsB_10–23_–modified peptides, but not to IA^g7^ tetramers containing irrelevant peptides human CLIP_87–101_ or hen egg lysozyme_11–25_ (HEL_11–25_) (Figure 1B). To further confirm specificity, we used fluorochrome-conjugated 1B2 to stain peptide-pulsed bone marrow–derived DCs (BMDCs). BMDCs pulsed with InsBP8E and, to a lesser extent, P8G showed a significant increase in 1B2 geometric MFI (gMFI) compared with BMDCs pulsed with vehicle control (Figure 1, C and D). Using biolayer interferometry, we confirmed a preference for InsBP8E over InsBP8G, where the relative affinity (*K*_D_) of 1B2 for InsBP8E:IA^g7^ was nearly 3-fold that of InsBP8G:IA^g7^ (8.5 × 10^–9^M versus 2.7 × 10^–8^M, respectively).

To further characterize antibody specificity, we next determined whether the 1B2 antibody could block CD4^+^ T cell responses to endogenous, islet-derived antigen. InsB_10–23_-specific TCR-transgenic 8F10 T cells were cocultured with NOD splenocytes pulsed with islets or peptides in the presence of 1B2 or IgG1 isotype control antibody (28). The 8F10 CD4^+^ T cells proliferated in response to both InsB_10–23_ and InsBP8G peptides and, to a lesser extent, NOD islets at a level similar to that shown in previously published data (28) (Figure 1, E and F). Addition of 1B2 antibody significantly (*P* < 0.001) reduced 8F10 CD4^+^ T cell proliferation in response to all 3 stimuli (InsB_10–23_, InsBP8G, and islets).

In another test of 1B2 specificity, we used the AS150 T cell hybridoma, which is specific for InsB_10–23_ peptide and secretes IL-2 in response to InsBP8E-pulsed antigen-presenting cells (APCs) (33, 36). At low-peptide concentrations, this response was inhibited with the addition of 1B2 antibody (Supplemental Figure 1; supplemental material available online with this article; https://doi.org/10.1172/JCI168601DS1). In contrast, 1B2 had no effect on BDC2.5 CD4^+^ T cell proliferation in response to the cognate antigen 2.5 hybrid-peptide (2.5HP), a fusion peptide composed of insulin C and chromogranin A (Supplemental Figure 2). These data demonstrate that the 1B2 antibody is specific for the InsB_10–23_:IA^g7^ peptide-MHC complex. Importantly, its affinity is sufficient to inhibit cognate T cell activation and proliferation in the presence of naturally processed and presented InsB_10–23_ antigen.

### 1B2 CAR Tregs are stable and retain specificity for InsB_10–23_.

**Figure 2:**
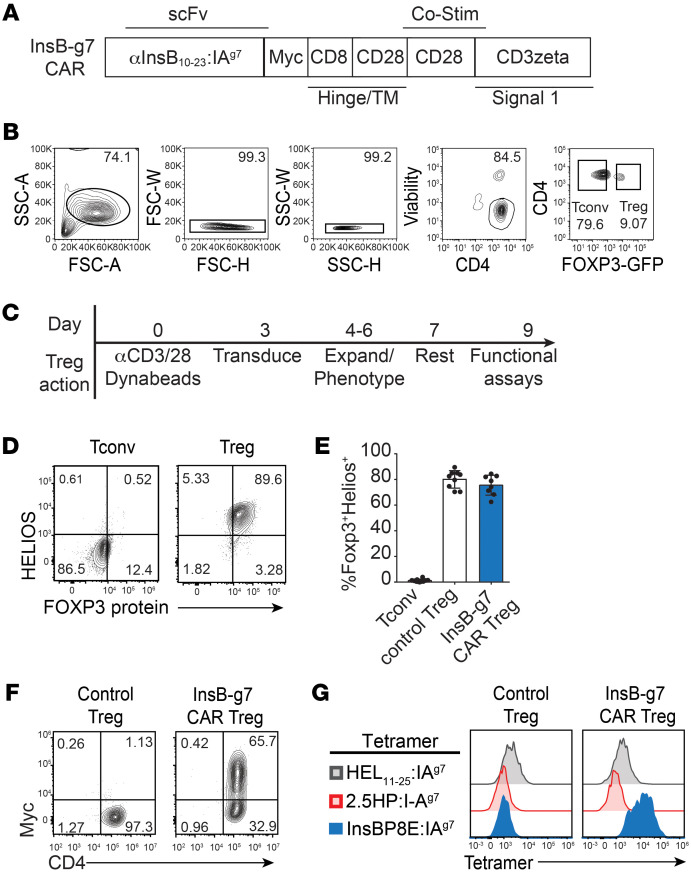
In vitro–expanded InsB-g7 CAR Tregs retain FOXP3 expression and bind cognate antigen. (**A**) Schematic depicting the design of InsB-g7 CAR derived from the variable region of the 1B2 antibody. TM, transmembrane. (**B**) Representative flow cytometry plots showing gating strategy used to sort GFP^+^ Tregs from NOD.*Foxp3^EGFP^* mice. (**C**) Timeline of protocol used to engineer and expand CAR Tregs from NOD mice. (**D**) Representative flow cytometry plots showing purity of sorted and expanded GFP^+^ Tregs compared with GFP^–^ Tconvs. Cells are gated on size, viability, and CD4^+^ T cells. (**E**) Quantification of the purity of control Tregs and InsB-g7 CAR Tregs compared with expanded GFP^–^ Tconvs. Data are pooled from 8–9 experiments, with each data point representing 1 individual experiment. (**F**) Representative flow cytometry plots showing CAR expression, as assessed by Myc tag staining, in InsB-g7 CAR Tregs compared with control Tregs. (**G**) Tetramer staining of Myc^+^ InsB-g7 CAR Tregs compared with Myc^–^ untransduced control Tregs. Data are representative of 9 experiments.

We next converted the 1B2 mAb into a CAR by linking heavy- and light-chain variable domain sequences from the 1B2 antibody and cloning upstream of an extracellular Myc-epitope tag followed by CD28 and CD3ζ intracellular signaling domains (17) (Figure 2A). To characterize the specificity of the resulting InsB-g7 CAR, Tregs were sorted from NOD.*Foxp3*^EGFP^ reporter mice and transduced with retrovirus encoding the InsB-g7 CAR or control (Figure 2, B and C). Compared with GFP^neg^ conventional T cells (Tconvs), sorted CAR Tregs were more than 90% FOXP3^+^, and the majority coexpressed HELIOS, another transcription factor characteristic of the Treg lineage (Figure 2, D and E). Furthermore, InsB-g7 CAR Tregs bound the InsBP8E:IA^g7^ tetramer, but not the 2.5HP:IA^g7^ tetramer (Figure 2, F and G). Collectively, these data demonstrate that, when reformatted as a CAR, the 1B2 antibody scFv had preserved insulin peptide–MHC complex specificity and that NOD Treg specificity could be redirected toward an islet antigen upon expression of this CAR.

### InsB-g7 CAR T cells are activated by synthetic and naturally presented insulin peptides.

**Figure 3:**
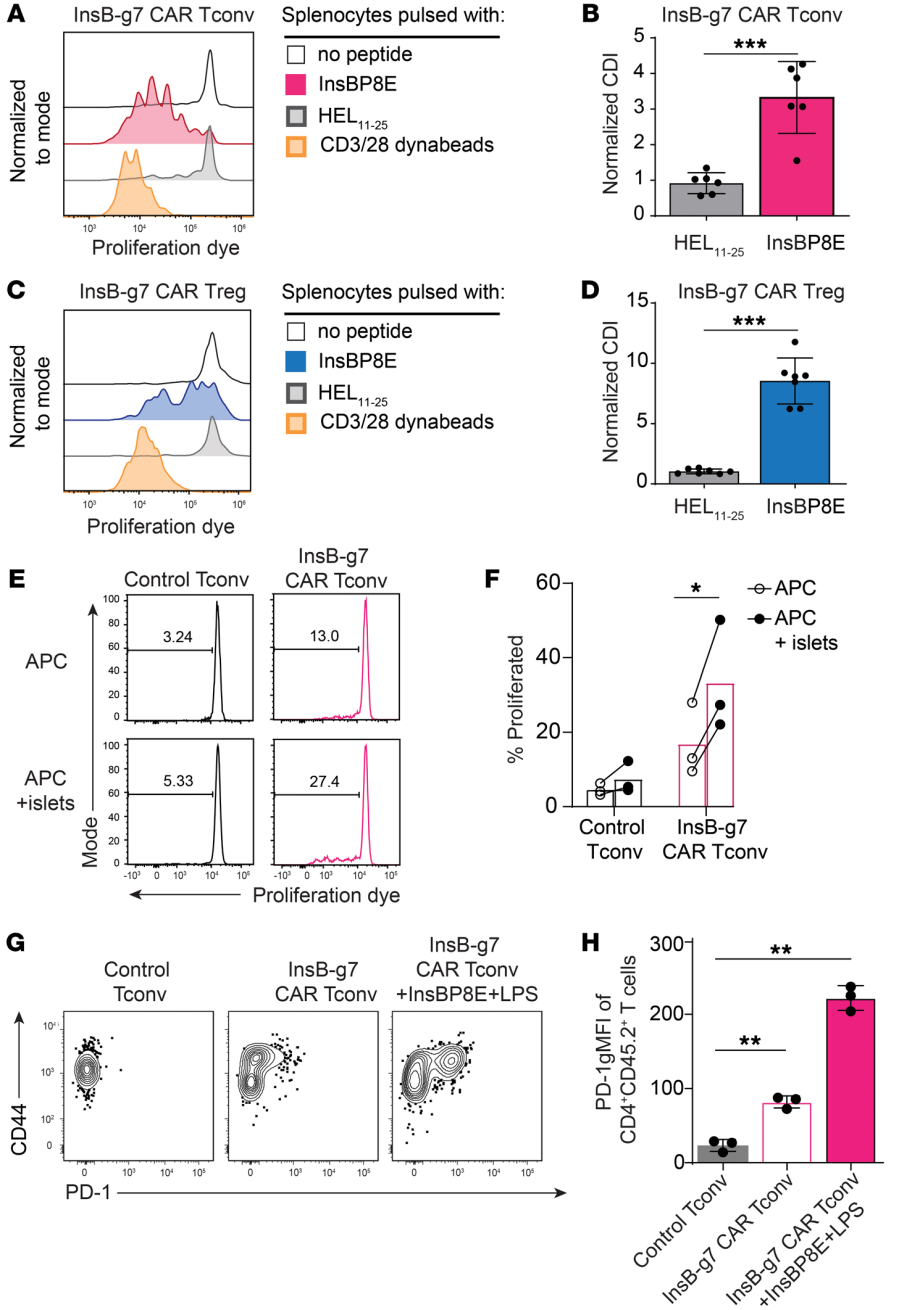
InsB-g7 CAR T cells are activated by synthetic and naturally presented insulin peptides. (**A**) Representative flow cytometry histograms showing in vitro proliferation of InsB-g7 CAR Tconvs following 3-day coculture with anti-CD3/CD28 antibody-coated Dynabeads for splenocytes pulsed with no peptide, InsBP8E, or HEL_11–25_. (**B**) Quantification of cumulative division index (CDI) of data shown in **A**. Data are from 6 independent experiments. *n* = 6/group. One-way ANOVA with multiple comparisons. ***P < 0.001. (**C**) Representative flow cytometry plots showing in vitro proliferation of InsB-g7 CAR Tregs following 3-day coculture with splenocytes pulsed with no peptide, InsBP8E, HEL_11–25_–, or CD3/CD28-coated Dynabeads. (**D**) Quantification of **C**. Data are from 7 independent experiments. *n* = 7/group. One-way ANOVA with multiple comparisons. ***P < 0.001. (**E**) Representative flow cytometry histograms showing untransduced control Tconv and InsB-g7 CAR Tconv proliferation after 3-day coculture with APCs alone or together with NOD islets. (**F**) Quantification of **E**. Data are pooled from 3 experiments. Two-way ANOVA with multiple comparisons. *P < 0.05. (**G**) Representative flow cytometry plots showing PD-1 expression on CD4^+^CD45.2^+^ untransduced control Tconvs and InsB-g7 CAR Tconvs isolated from the spleen 7 days after transfer into 8-week-old female NOD mice. One of the treatment groups received InsBP8E peptide with LPS intravenously on day 0. (**H**) Quantification of PD-1gMFI of cells depicted in **G**. Data are representative of 2 independent experiments. *n* = 3 per group. One-way ANOVA with multiple comparisons. **P < 0.01.

To further test the specificity and function of the InsB-g7 CAR, we assessed the proliferative response of InsB-g7 CAR T cells to InsB_10–23_ peptide. 1B2 CAR Tconvs or Tregs were labeled with a proliferation dye and cocultured in vitro with irradiated NOD splenocytes in the presence of an irrelevant peptide control (HEL_11–25_) or InsB P8E. In response to InsBP8E peptide, both InsB-g7 CAR Tconvs and Tregs underwent significant proliferation (Figure 3, A–D). Consistent with the hypoproliferative nature of Tregs (37), InsB-g7 CAR Tconvs proliferated more extensively in response to InsBP8E than InsB-g7 CAR Tregs, but the cumulative division index (CDI) was greater in InsB-g7 CAR Tregs due to lower background proliferation in the absence of exogenous antigen.

We next tested to determine whether the InsB-g7 CAR could stimulate T cell activation in response to islet-derived antigen. These experiments were done with Tconvs due to their higher in vitro proliferative capacity compared with Tregs. Control Tconvs or InsB-g7 CAR Tconvs were labeled with a proliferation dye and cocultured in vitro with T cell–depleted NOD splenocytes in the presence or absence of NOD islets. A substantial fraction of InsB-g7 CAR Tconvs (~30%) proliferated in response to islets, whereas control Tconvs did not (Figure 3, E and F). Proliferation by InsB-g7 CAR Tconvs in the absence of islets could be due to high levels of insulin in the ImmunoCult XF culture medium, measured to be approximately 7mg/ml, and/or the fact that NOD-derived APCs maybe naturally loaded with insulin.

To determine whether InsB-g7 CAR Tconvs could respond to endogenous islet-derived antigen in vivo, control or InsB-g7 CAR Tconvs were injected into 8-week-old female NOD mice and 7 days later, the T cells were isolated from the secondary lymphoid organs and analyzed by flow cytometry for PD-1 expression as an indirect measure of stimulation (38). Whereas control Tconvs had no detectable PD-1 expression, approximately 20% of InsB-g7 CAR Tconvs expressed PD-1. As a positive control, mice were immunized with InsBP8E and LPS, resulting in nearly 60% of 1B2 CAR Tconvs staining positive for PD-1 (Figure 3, G and H). These data show that the InsB-g7 CAR can detect islet-derived antigen in vivo and, similar to what was observed with TCR ligation, become activated and upregulate PD-1.

### InsB-g7 CAR Tregs mediate bystander suppression in vitro.

**Figure 4:**
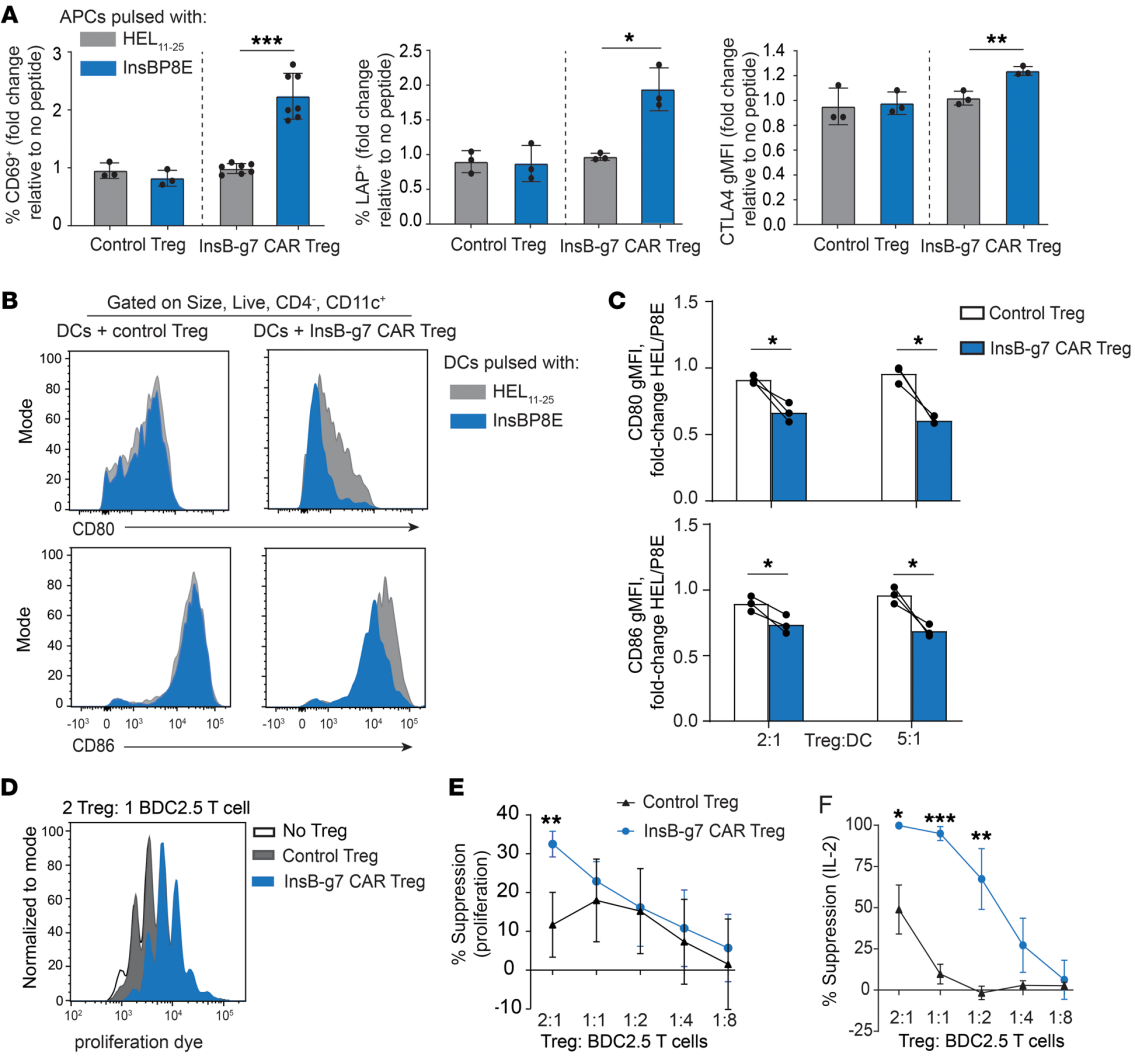
InsB-g7 CAR Tregs mediate bystander suppression in vitro. (**A**) Quantification of flow cytometry data showing the fold change in the frequency of Tregs expressing CD69 (left) and LAP (middle) and gMFI of CTLA-4 (right) expression on InsB-g7 CAR Tregs or untransduced control Tregs after overnight coculture with splenocytes pulsed with InsBP8E or HEL_11–25_ relative to cells cocultured in the absence of peptide. Data are from at least 3 independent experiments. *n* = 3–7/ group. One-way ANOVA with multiple comparisons. *P < 0.05; **P < 0.01; ***P < 0.001. (**B**) Representative histograms showing CD80 (top) and CD86 (bottom) expression on HEL_11–25_– or InsBP8E–pulsed splenic CD11c^+^ DCs following 2-day coculture with control Tregs or InsB-g7 Tregs at a ratio of 2:1 Tregs/DCs. (**C**) Quantification of data in **B** represented as fold change in CD80 (top) or CD86 (bottom) gMFI of DCs pulsed with HEL_11–25_ relative to InsBP8E. Data are pooled from 3 independent experiments. Paired 2-tailed *t* test. *P < 0.05. (**D**) Representative flow cytometry data from in vitro Treg-suppression assays showing proliferation of BDC2.5 T cells in the presence of antigen-loaded DCs (pulsed with 10 nM p63 and 10 μM insulin P8E peptides) and control Tregs or InsB-g7 CAR Tregs. (**E**) Quantification of BDC2.5 T cell proliferation from the experiment described in **D**. (**F**) Quantification of IL-2 cytokine in the culture supernatants from the experiment described in **D**. Data in **E** and **F** are normalized to no-Treg group and are from 2 independent experiments. *n* = 3/group. Two-way ANOVA with multiple comparisons of control Treg group to InsB-g7 CAR Treg group at each Treg/T cell ratio. *P < 0.05; **P < 0.01; ***P < 0.001.

After confirming that InsB-g7 CAR Tregs were stimulated by islet-derived antigen, we next assessed their antigen-stimulated suppressive capacity. We first tested to determine whether CAR stimulation induced expression of proteins related to Treg activation and suppression. Control Tregs and InsB-g7 CAR Tregs were cocultured in vitro overnight in the presence of NOD splenocytes with either InsBP8E, HEL_11–25_, or no peptide. We found that InsBP8E stimulation led to a significant increase in CD69, LAP, and CTLA4 expression on InsB-g7 CAR Tregs compared with HEL_11–25_-stimulated InsB-g7 CAR Tregs; no effect was observed in control Tregs, regardless of peptide antigen (Figure 4A). Another in vitro correlate of in vivo suppressive function is antigen-stimulated transendocytosis of costimulatory molecules CD80 and CD86 from APCs, which we have found to best predict in vivo CAR Treg function (39). When InsB-g7 CAR Tregs were cocultured with splenic CD11c^+^ DCs pulsed with InsBP8E, both CD80 and CD86 expression on DCs was significantly reduced compared with that on DCs pulsed with HEL_11–25_ (Figure 4, B and C). In contrast, there was no change in CD80/86 expression on DCs pulsed with InsBP8E in the presence of control Tregs (Figure 4, B and C). These data show that stimulation of InsB-g7 CAR Tregs with cognate antigen results in increased expression of molecules associated with Treg suppression and facilitates enhanced transendocytosis of CD80 and CD86 from APCs.

We next determined whether CAR stimulation enhanced Treg suppression in vitro using BDC2.5 T cells as responders. To maximize Treg-mediated effects on expression of CD80 and CD86, InsB-g7 CAR or control Tregs were cocultured overnight with DCs in the presence of the peptides InsBP8E and P63, a mimotope for the BDC2.5 TCR. The next day, CD4^+^ BDC2.5 T cells were added to Treg/DC cultures and BDC2.5 T cell proliferation was assessed by flow cytometry after 3 additional days. Compared with the no-Treg control, both control Tregs and InsB-g7 CAR Tregs suppressed BDC2.5 T cell proliferation and IL-2 production (Figure 4, D–F). However, at the 2:1 Treg/T cell ratio, the InsB-g7 CAR Tregs were significantly more suppressive than control Tregs. Moreover, InsB-g7 CAR Tregs suppressed IL-2 production significantly more than control Tregs at multiple ratios (Figure 4, D–F). Collectively, these data show InsB-g7 CAR stimulation leads to increased expression of markers of activation and suppression, resulting in the suppression of DCs and effector function of T cells with disparate antigen specificity.

### InsB-g7 CAR Tregs suppress BDC 2.5 T cell–induced autoimmune diabetes.

**Figure 5:**
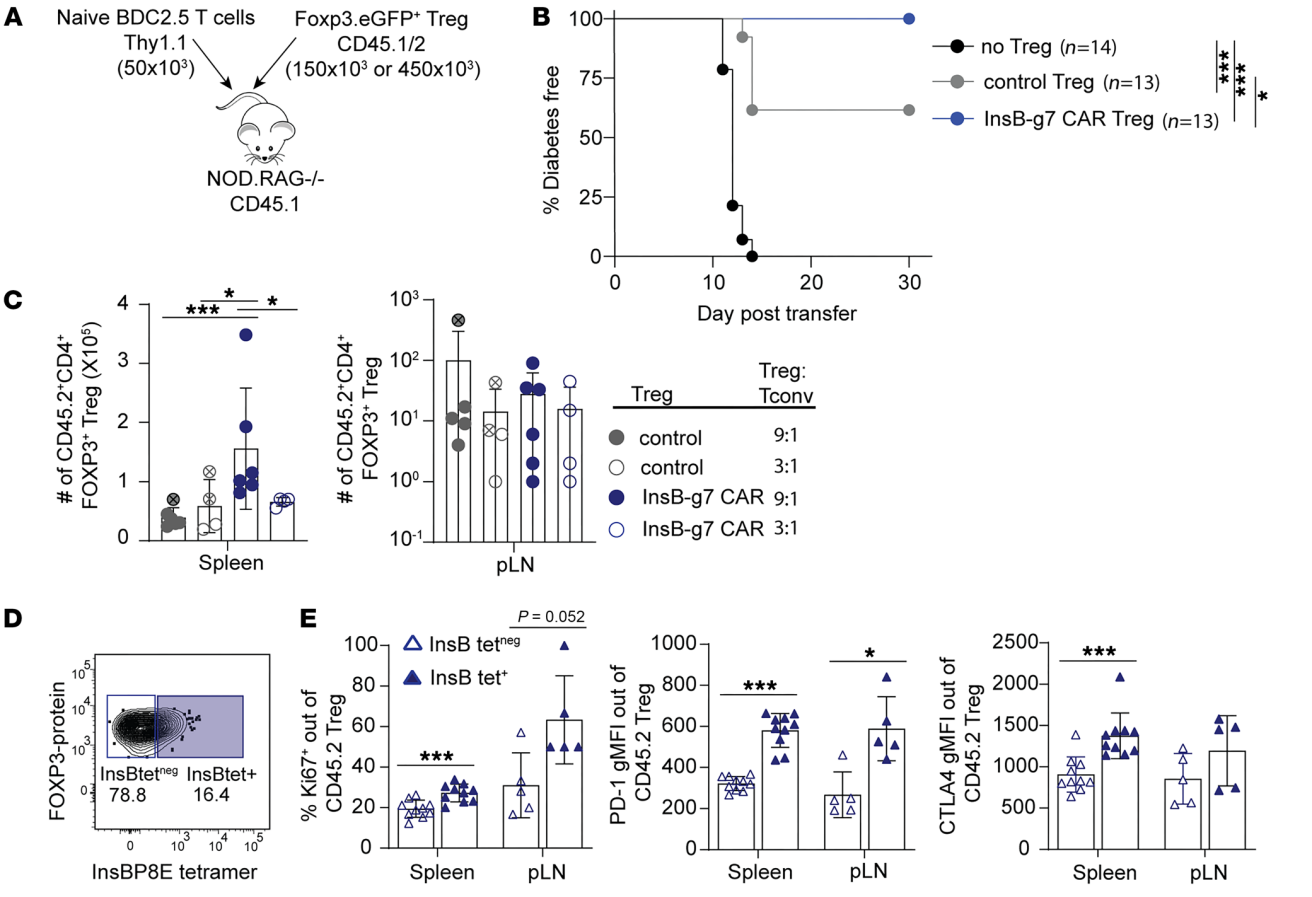
InsB-g7 CAR Tregs suppress BDC 2.5 T cell–induced autoimmune diabetes. (**A**) Experimental design indicating that 450 × 10^3^ (9:1) or 150 × 10^3^ (3:1) untransduced control Tregs or InsB-g7CAR Tregs were cotransferred with 50 × 10^3^ naive BDC2.5 CD4^+^ T cells into 6- to 10-week-old NOD.*Rag1^–/–^* recipient mice. (**B**) Diabetes-free survival of mice described in **A** with pooled 9:1 and 3:1 Treg/BDC T cell treatment groups. Data are from 2–3 independent experiments. No Tregs, *n* = 14; 150 × 10^3^ control Tregs, *n* = 4; 450 × 10^3^ control Tregs, *n* = 9; 150 × 10^3^ 1B2 Tregs, *n* = 4; 450 × 10^3^ 1B2 Tregs, *n* = 9. Log-rank test with Bonferroni’s correction. *P < 0.05; ***P < 0.001. (**C**) Quantification of CD4^+^CD45.2^+^FOXP3^+^ Tregs isolated from spleen and pLNs from mice treated at 9:1 (filled circles) or 3:1 (open circles) Treg/Tconv, as indicated in pooled groups shown in **B**. Circles containing crosshairs depict diabetic mice, with all mice analyzed either 2 days after becoming diabetic or at day 30 after transfer. Two-way ANOVA with multiple comparisons. *P < 0.05; ***P < 0.001. (**D**) Representative flow cytometry plot illustrating gating strategy for identification of CD4^+^CD45.2^+^ InsBP8E tetramer^+^ and tetramer^neg^ FOXP3^+^ Tregs from InsB-g7 CAR Treg–treated mice shown in **B**. (**E**) The frequency of Ki67^+^ and gMFI of surface PD-1 and intracellular CTLA4 of tetramer^neg^ and tetramer^+^ InsBg7 CAR Tregs. Data are pooled from mice treated at 9:1 and 3:1 InsB-g7 CAR Tregs/BDC2.5 T cells and are from 2–3 independent experiments. *n* = 5–10 mice/group. Only mice with more than 10 CD4^+^CD45.2^+^ T cells (limit of detection) were included in the analysis. Student’s *t* test, 2-tailed. *P < 0.05; ***P < 0.001.

To test the in vivo function of InsB-g7 CAR Tregs, we first used an adoptive transfer model of autoimmune diabetes. In this model, the Teff/Treg ratio can be controlled and antigen encounter synchronized. We transferred 50 × 10^3^ naive BDC2.5 CD4^+^ T cells alone or together with either untransduced control Tregs or InsB-g7 CAR Tregs at a 3:1 or 9:1 Treg/BDC2.5 T cell ratio into NOD.*Rag1^–/–^* recipient mice (Figure 5A). Prior to adoptive transfer, Tregs expressed high levels of FOXP3 compared with sorted, GFP^neg^ Tconvs, and more than 90% of InsB-g7 CAR Tregs bound to the InsBP8G tetramer, demonstrating high Treg purity and CAR expression (Supplemental Figure 3). Mice that were given BDC2.5 T cells alone developed diabetes within 14 days, whereas mice coinjected with InsB-g7 CAR Tregs were completely protected from autoimmune disease (Figure 5B). Similarly, and in agreement with previously published data (15), mice treated with in vitro–expanded BDC2.5 Tregs at a 3:1 Treg/BDC2.5 T cell ratio were also completely protected from disease (Supplemental Figure 4). In contrast, the InsB-g7 CAR Tregs were significantly (*P* < 0.05) better at preventing BDC2.5 T cell–induced disease compared with control Tregs: 3 of 9 mice and 2 of 4 mice coinjected with control Tregs at 9:1 and 3:1 Treg/BDC2.5 T cell ratios, respectively, developed diabetes.

To gain more insight into how InsB-g7 CAR Tregs suppressed autoimmune diabetes, we used flow cytometry to enumerate and phenotype the CAR Tregs and BDC2.5 T cells either within 2 days of disease onset or, for those mice that remained nondiabetic, 30 days after cell transfer. Using CD45.2 as a congenic marker to identify the CAR Tregs, we found that the spleens of mice receiving the InsB-g7 CAR Tregs at a 9:1 ratio (Treg/BDC2.5) contained significantly more CD45.2^+^ Tregs than those of mice receiving InsB-g7CAR Tregs at a 3:1 ratio or mice that received control Tregs (Figure 5C). We next determined whether protection from autoimmune disease in InsB-g7 CAR Treg–treated mice was associated with enhanced InsB-g7 CAR Treg proliferation and suppressive phenotype. While nearly all CD45.2^+^ cells retained FOXP3 expression, only a subset maintained surface expression of the InsB-g7 CAR, as assessed by InsBP8E tetramer staining (Figure 5D). Comparing the phenotype of InsBP8E tetramer^+^ and tetramer^neg^ cells, the tetramer^+^ cells showed higher expression of Ki67 in both the spleen and pancreatic LNs (pLNs) (Figure 5E). Furthermore, the tetramer^+^ InsB-g7 CAR Tregs also showed significantly higher levels of PD-1 and CTLA4 (Figure 5E), showing that InsB-g7 CAR Tregs were stimulated in vivo. Thus, InsB-g7 CAR Treg–treated mice had the highest numbers of CAR Tregs in the spleen and the lowest incidence of autoimmune diabetes.

### InsB-g7 CAR Tregs reduce the number of BDC 2.5 T effector cells in peripheral lymphoid organs and pancreas.

**Figure 6:**
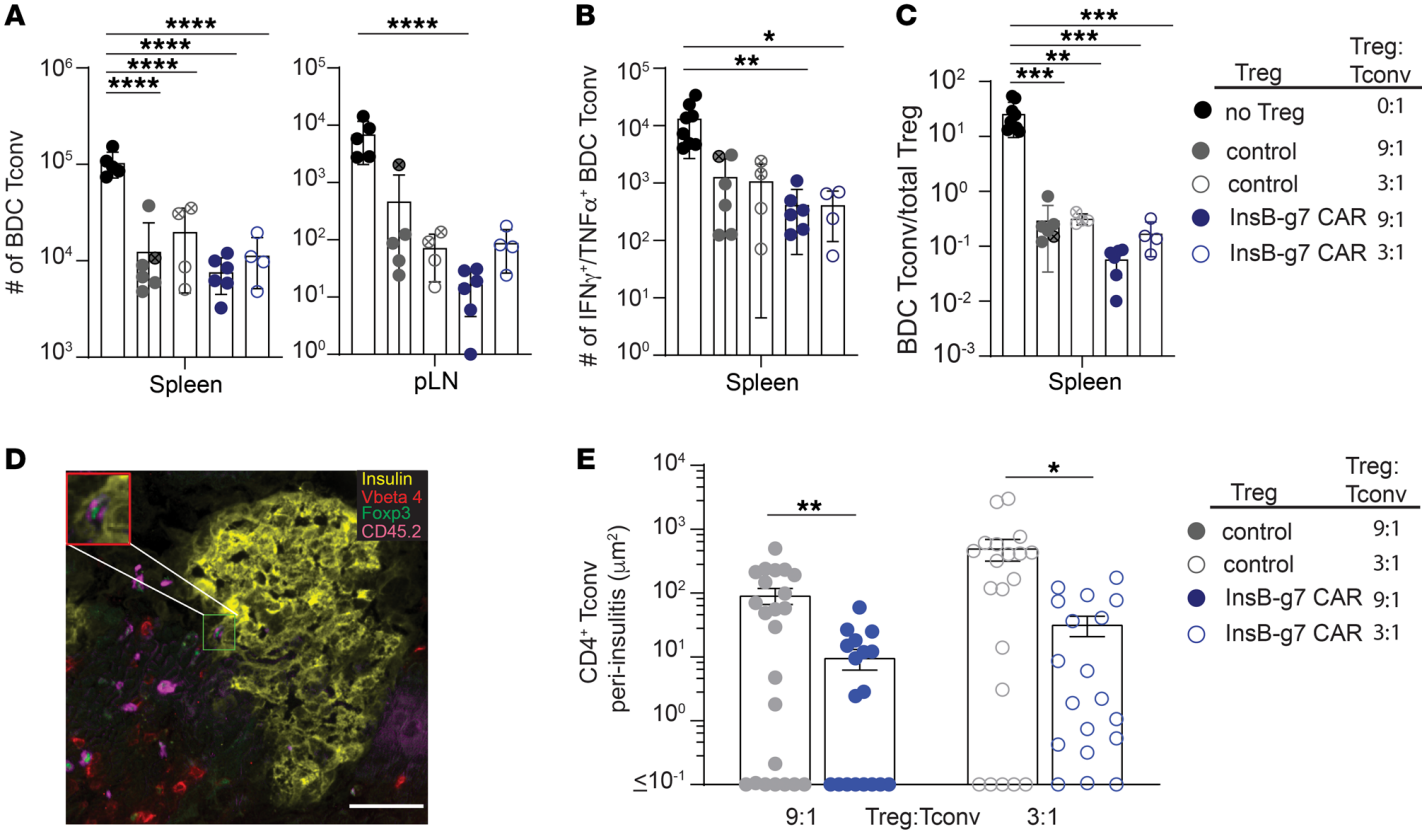
InsB-g7 CAR Tregs reduce the number of BDC 2.5 T effector cells in peripheral lymphoid organs and insulitis. (**A**) Quantification of the number of BDC2.5 Tconvs in the spleen and pLNs from mice described in Figure 5, A and B. ****P < 0.0001. (**B**) Number of IFN-γ^+^TNF-α^+^ BDC2.5 Tconvs from spleen of mice described in Figure 5, A and B, and treated at 9:1 or 3:1 Tregs/Tconvs. (**C**) Ratio of CD4^+^Thy1.1^+^FOXP3^neg^ BDC Tconvs/CD4^+^CD45.2^+^FOXP3^+^ Tregs from mice described in Figure 5, A and B. Data are from 2–3 independent experiments. *n* = 4–14 mice/group. One-way ANOVA with multiple comparisons. *P < 0.05; **P < 0.01; ***P < 0.001. (**D**) Representative epifluorescence image of a histological section of the pancreas from a mouse described in Figure 5, A and B, at day 30 after treatment with InsB-g7 CAR Tregs at 3:1 Tregs/Tconvs. Scale bar: 50 μm. Inset shows CD45.2^+^ (magenta), FOXP3^+^ (green) InsB-g7 CAR Tregs and TCR Vβ4^+^ (red) BDC2.5 T cells within the insulin^+^ (yellow) peri-islet cellular infiltrate. Original magnification (inset): ×1.37. (**E**) Quantification of total area occupied by FOXP3^neg^CD45.2^neg^TCR Vβ4^+^CD4^+^ Tconvs within the peri-islet infiltrate of mice shown in **B** analyzed either 2 days after becoming diabetic or at day 30 after transfer. Data are from 3–4 mice/group with 6–10 images/mouse and 88 total images. Kruskal-Wallis test. *P < 0.05; **P < 0.01.

We next examined the effects of InsB-g7 CAR Tregs on BDC2.5 T cell expansion and/or effector cytokine production. All Treg-treated mice had significantly reduced numbers of BDC2.5 T cells in the spleen, whereas mice treated with InsB-g7 CAR Tregs at a 9:1 Treg/Tconv ratio also had a significantly reduced number of BDC2.5 T cells in the pLN (Figure 6A). In addition to reduced BDC2.5 T cell numbers, InsB-g7 CAR Treg treatment led to a significant reduction in the number of IFN-γ– and TNF-α–producing polyfunctional BDC2.5 T effector cells in the spleen (Figure 6B). While all mice receiving Tregs had a significant reduction in the BDC Tconv/Treg ratio, mice receiving the highest dose of InsB-g7 CAR Tregs had the lowest ratio of BDC Tconvs/Tregs (Figure 6C). The lack of a significant difference in BDC2.5 T cell numbers between mice receiving control and InsB-g7 CAR Tregs may be due to the lymphophenic environment of the *Rag1^–/–^* host, which causes the BDC2.5 T cells to undergo antigen-independent homeostatic proliferation, a phenomenon known to be well controlled by polyclonal Tregs (40). Nevertheless, using immunofluorescent staining of pancreas tissue either at the time of diabetes diagnosis or 30 days after transfer, we found FOXP3^+^ InsB-g7 CAR Tregs in close islet proximity, in peri-insulitis, and within islets (Figure 6D). Histologically there was a significant difference in the amount of BDC2.5 T cell insulitis in the pancreas of mice treated with InsB-g7 CAR Tregs (Figure 6, D and E). At both the 9:1 and 3:1 Treg/BDC2.5 T cell ratios, mice treated with InsB-g7 CAR Tregs showed a significant, nearly 10-fold reduction in the total area around the islets that was occupied by BDC2.5 Tconvs (9:1, *P* < 0.01; 3:1, *P* < 0.05). In summary, InsB-g7 CAR Tregs likely prevent autoimmunity via suppression of BDC2.5 T effector function.

### InsB-g7 CAR Tregs prevent spontaneous autoimmune diabetes.

**Figure 7:**
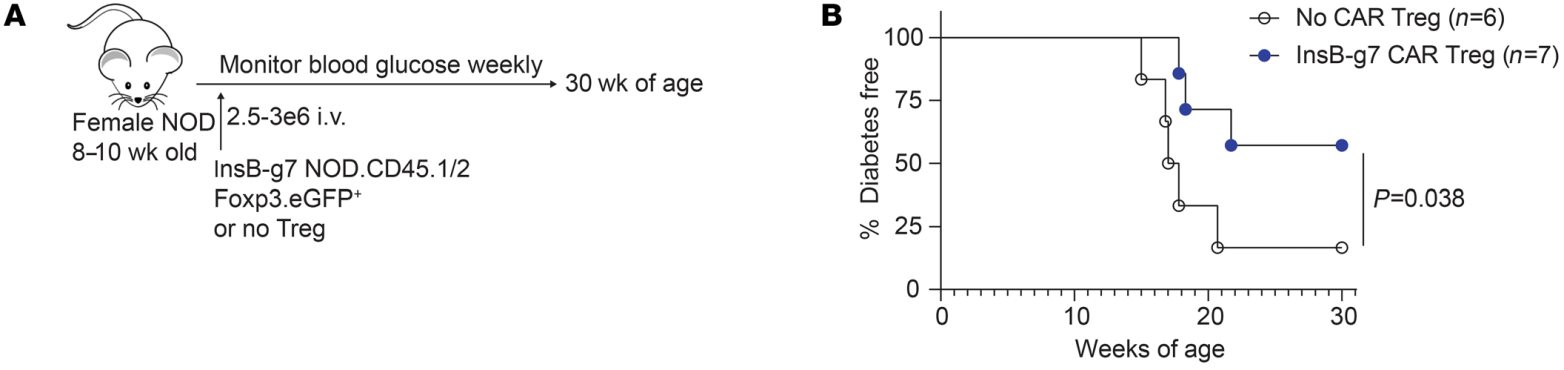
InsB-g7 CAR Tregs prevent spontaneous autoimmune diabetes. (**A**) Experimental design in which 8- to 10-week-old NOD female mice received 2.5–3 × 10^6^ InsB-g7 CAR Tregs or no cells and were monitored until 30 weeks of age for the development of spontaneous diabetes. (**B**) Spontaneous diabetes–free survival of mice described in **A**. Data are pooled from 4 independent experiments. *n* = 6–7mice/group. Gehan-Breslow-Wilcoxon test.

We next treated WT NOD mice with InsB-g7 CAR Tregs to determine effects on prevention of spontaneous autoimmune diabetes in an immunocompetent model. InsB-g7 CAR Tregs or vehicle control were transferred into nondiabetic 8- to 10-week-old NOD female mice (Figure 7A). At this age, most female NOD mice have autoantibodies and substantial insulitis, similarly to stage 1 disease in humans (30). Control mice that received vehicle alone developed diabetes at rates consistent with our colony’s historical incidence, with approximately 80% diabetic by 30 weeks of age. In contrast, mice treated with a single injection of InsB-g7 CAR Tregs were significantly protected from diabetes, with only 3 of 7 mice (43%) developing disease by 30 weeks of age (Figure 7B). Despite the significant reduction in autoimmune disease incidence in InsB-g7 CAR Treg–treated mice, at the experimental end point of 30 weeks, we could not detect donor CD45.2^+^ cells in the secondary lymphoid organs (including pLN) in either diabetic or nondiabetic mice (data not shown).

## Discussion

In this study, we show that the specificity of Tregs can be redirected toward an islet-derived antigen through the expression of a TCR-like peptide-MHCII–specific CAR. Expression of the resulting islet antigen–specific CAR in Tregs led to enhanced proliferation in response to islet-derived antigen and suppression of effector T cell proliferation and cytokine production in vitro. In vivo, CAR Tregs preferentially accumulated in the pancreas-draining LNs, where they upregulated molecules associated with activation, resulting in the suppression of effector T cell proliferation, cytokine production, and ultimately, autoimmune diabetes. The potential of TCR-like CARs specific for peptides presented on MHCI is increasingly appreciated in cancer (41, 42). To the best of our knowledge, this is the first example of the successful use of TCR-like CAR specific for a peptide-MHCII complex to control an autoimmune process.

There are a few recent reports on the use of CAR T cells for the treatment of autoimmune diabetes. In one study, an antibody (mAb287) with specificity similar to that of 1B2 was used to generate a TCR-like CAR and redirect CD8^+^ T cells to kill APCs presenting InsB in the context of IA^g7^ (43). Although these CD8^+^ CAR T cells delayed diabetes onset in NOD mice, protection declined with time and no significant difference in the overall incidence was observed by 30 weeks of age. Since many different APCs and islet-derived antigens are implicated in autoimmune pathogenesis (44–46), eliminating APCs presenting only 1 antigen may not be sufficiently potent to prevent activation of diabetogenic T cells. In terms of CAR Tregs, enforced FOXP3 expression in Tconvs or coexpression of an antiinsulin scFv-CAR (25) has been explored. Although the antiinsulin CAR Tregs were suppressive in vitro, they did not prevent spontaneous autoimmune diabetes in NOD mice, possibly because of low CAR avidity for antigen.

Considering evidence for lack of single-peptide specificity among TCRs (47), we reasoned that an advantage of using a TCR-like scFv CAR rather than a TCR could be increased specificity and affinity. How TCR and CAR affinity/avidity affects Treg activity remains an open question. There are varying reports of Tregs engineered to express islet antigen–specific TCRs. Human TCR Tregs specific for InsB_11–30_ in the context of HLA-DR3 were significantly less suppressive than influenza hemagglutinin–specific Tregs, but the suppression could be enhanced either through modification of islet antigen presentation or use of higher affinity mimotopes (48). When TCR Tregs with shared specificity for glutamic acid decarboxylase were compared, cells expressing TCRs with relatively high affinity were more suppressive than lower-affinity cells (49). Furthermore, TCR transgenic BDC2.5 Tregs are potently suppressive in vitro and in vivo and have higher 2D affinity (1.8 × 10^–3^ μM^4^) and functional avidity than the well-characterized SMARTA CD4^+^ T cells, which are specific for foreign antigen gp_66–77_:IA^b^ (7.3 × 10^–4^μM^4^) (15, 16, 50). In contrast, when Tconvs were engineered to express FOXP3 and TCRs of varying avidity for IGRP and preproinsulin, functional avidity negatively correlated with suppression (14). Given the lack of consensus on how antigen receptor affinity relates to Treg-suppressive function, an interesting future direction would be to compare the functional effects of modified 1B2 scFv CAR with varying affinities as well as CARs with modified intracellular signaling domains predicted to mediate stronger versus weaker activation signals (51).

All past studies of Tregs in models of autoimmune diabetes used BDC2.5 Tregs to show that antigen-specific Tregs were more suppressive than polyclonal Tregs (13–16). In BDC2.5 T cell–induced diabetes models, BDC2.5 Tregs prevent disease at ratios as low as 1 Treg/1 T cell (14, 15). We found ratios of 3 InsB-g7 CAR Tregs/1 BDC T cell were required for complete protection from autoimmune diabetes. Since Tregs can suppress effector T cell responses by removing cognate p:MHCII from the surface of APCs via trogocytosis (52), suppression of T cells with shared antigen specificity is likely greater compared with the bystander T cell suppression being tested in our model. In support of this possibility, GAD86 TCR transgenic Tregs did not protect mice from BDC2.5-induced diabetes when given at 1:1 Treg/BDC T cell ratios (15). These data suggest that CAR or TCR Treg therapeutic efficacy can be influenced by TCR or CAR affinity for antigen and/or the context of antigen presentation (e.g., direct versus bystander suppression).

In terms of effects in the NOD spontaneous model, multiple studies showed a lack of effect of polyclonal Tregs, but positive effects of BDC2.5 Tregs. For example, injection of 2 × 10^6^ BDC2.5 Tregs prevented islet graft rejection and spontaneous autoimmune diabetes, whereas 5 10^6^ polyclonal Tregs had no effect (15). Furthermore, 8 × 10^6^ polyclonal Tregs failed to inhibit spontaneous diabetes development in NOD mice while 2 × 10^6^ P31-peptide expanded Tregs (which have specificity similar to that of BDC2.5 TCRs) attenuated disease development in 60% of animals (53). Similarly, at least 3 times more polyclonal nonspecific DC-expanded Tregs were required to prevent spontaneous diabetes in NOD mice compared with DC-expanded BDC2.5 Tregs (16). The numbers of InsB-g7 CAR Tregs used in our study to prevent spontaneous autoimmune disease are in line with these previous studies, overall highlighting the potency of antigen-specific Tregs to prevent disease in NOD mice compared with nonspecific polyclonal Tregs not selected for islet specificity.

Tregs mediate suppression by a number of mechanisms, including production of inhibitory cytokines, metabolic disruption, expression of coinhibitory molecules, modulating APC function, competition for antigen or cytokines, or direct cytolysis. Evidence that InsB-g7 CAR Tregs potently suppress IL-2 production suggests that consumption of IL-2 could contribute to a negative feedback loop to prevent autoimmunity (54). Further, antigen stimulation of InsB-g7 CAR Tregs resulted in elevated expression of CTLA-4 and significantly reduced CD80 and CD86 expression on the surface of DCs, indicative of transendocytosis (39, 55, 56). Combined with evidence that InsB-g7 CAR Tregs suppress the proliferation and effector function of BCD2.5 T cells in vitro and in vivo, we speculate that InsB-g7 CAR Tregs function by diminishing costimulation from APCs presenting InsB_10–23_ antigen, thus resulting in bystander suppression of T cells with specificity for disparate islet antigens. Similar bystander suppressive function has recently been reported with TCR-engineered Tregs (14). If modulation of APC function through direct cell contact is a dominant mechanism of Treg-mediated suppression, this would favor the development of Treg therapies that target antigens on the surface of APCs rather than soluble antigens.

Treg homing to target tissue and draining LNs is required for optimal suppressive function (26). FOXP3^+^ insulin-specific CAR Tregs were detected in the spleen of NOD mice 4 months after a single injection of 2.5 × 10^6^ cells (25). In another study, DC-expanded BDC2.5 Tregs reversed autoimmune diabetes in NOD mice transplanted with islets, yet donor Tregs could not be detected in pLNs or pancreas 50 days after the first of 2 injections. Similarly, we could not find donor Tregs in the secondary lymphoid organs or pancreas of 30-week-old NOD mice, nearly 5 months after injection. Although CXCL10 produced in inflamed islets promotes the recruitment of CXCR3-expressing T cells, this cytokine network is not specific to the islets of Langerhans (57, 58). It could be that transient Treg-mediated suppression at the sites of antigen presentation in pLNs may be sufficient to prevent autoimmunity, but in cases where there is ongoing inflammation, it may be necessary for Tregs to access the pancreas.

Although the InsB-g7 CAR Tregs were greater than 80% CAR^+^ prior to adoptive transfer, we routinely observed a substantial reduction in CAR^+^ T cells among the CD45.2^+^ donor cell population within 1 week following transfer. Similarly, CAR T cells adapt to stimulation by downregulating CAR expression, with a positive correlation between CAR T cell avidity and the extent of CAR downregulation (59, 60). Thus there may be a need to balance CAR avidity with adaptation to levels of receptor expression to enhance therapeutic efficacy (59). Another factor limiting CAR expression could be anti-CAR antibodies, particularly in immunocompetent mice. In CD19^+^ CAR T cell trials, preexisting or therapy-induced antibodies against mouse single-chain variable fragments were observed, but how they affected therapeutic efficacy is not well understood (61). The 1B2 antibody was derived from the NOD mouse (32,) limiting the potential for immunity against single-chain variable fragments, although additional components of the CAR could be immunogenic. Notably, Tarbell et al. also found that injected BDC2.5 Tregs did not persist, so long-term Treg survival may not be necessary for tolerance induction (16).

Two important considerations for the use of p:MHC-specific CAR Tregs in a clinical context are the level of associated risks tolerated and when it would be feasible to treat with a cellular therapy. In terms of risks, despite the potential for destabilized FOXP3 expression, we never saw any overt pathology or acceleration of autoimmune diabetes in InsB-g7 CAR Treg–treated mice, suggesting that this therapy is well tolerated. Future work is required to understand the effect of these cells in mice that are already diabetic, since it would be difficult to envision the first application of engineered Tregs in prediabetic humans (as tested here in NOD mice). It is encouraging that it has been shown that BDC2.5 Tregs do have the potential to reverse disease in NOD mice that are already diabetic (15, 16).

In conclusion, here we have demonstrated the feasibility of using an InsB_10–23_-IA^g7^–specific mAb to create a peptide-MHCII–specific CAR. The use of InsB-g7 CAR Tregs for the reversal of autoimmune diabetes following islet transplant may be more amenable to translation compared with disease prevention and will be the focus of future studies. This work provides the first proof-of-concept, to our knowledge, that CAR Tregs have the potential to be used therapeutically in the context of T1D and in other organ-specific autoimmune or inflammatory disorders.

## Methods

### Mice.

NOD mice were purchased from Taconic. NOD.CD45.2 (catalog 014149), NOD.*Foxp3^EGFP^* (catalog 025097) (62), NOD.BDC2.5 TCR (catalog 004460), and NOD.*Rag1^–/–^* (catalog 003729) mice were purchased from The Jackson Laboratory. NOD.CD90.1 mice were generated by backcrossing BALB/cBy-Thy1a congenic onto NOD/ShiLtJ (catalog 001976) mice for 19 generations and maintained at the University of Minnesota. NOD.8F10 mice were a gift from Emil Unanue (Washington University, St. Louis, Missouri, USA) (28). NOD.CD45.2^Het^.*Foxp3^EGFP^* mice were generated by crossing NOD.CD45.2 mice with NOD.*Foxp3^EGFP^* mice, and F1 mice were used as Treg donors for experiments. NOD.CD90.1.BDC2.5 TCR mice were generated by crossing NOD.CD90.1 mice with NOD.BDC2.5 TCR mice to CD90.1 homozygosity. All mice were housed in specific pathogen–free conditions.

### 1B2 antibody staining of peptide-pulsed APCs and inhibition of T cell proliferation.

BMDCs were generated by culturing cells isolated from the femora and humeri of 6- to 8-week-old female NOD mice in RPMI 1640 (Thermo Fisher Scientific) supplemented with 10% fetal calf serum (Omega Scientific), 50 μM 2-mercaptoethanol (Thermo Fisher Scientific), 10 mM HEPES, penicillin/streptomycin (Thermo Fisher Scientific), and 20 ng/ml murine GM-CSF (PeproTech) for 10 days. For staining of peptide-pulsed cells, nonadherent cells were collected and incubated overnight in complete RPMI media containing 20 ng/ml GM-CSF, 1 mg/ml LPS (from *Escherichia col*i J5[Rc]; List Biological Laboratories), and 100 μM of peptide (GenScript) and then stained with 1B2-labeled with AF488 (Thermo Fisher Scientific) and surface antibodies against CD11b, CD11c, MHCII, and a live dead viability dye (Cytek Biosciences).

For 1B2-mediated inhibition of T cell proliferation, AS150 T cell hybridoma cells (originally generated in the laboratory of Emil Unanue and received as a gift from John Kappler, University of Colorado, Denver) were cultured for 24 hours in round-bottom 96-well plates (Corning) at 1:1 with NOD splenocytes in 200 ml complete RPMI media overnight in the presence of the indicated amount of peptide and 1B2 mAbs. For 1B2-mediated inhibition of 8F10 proliferation, splenocytes from NOD.8F10 TCR transgenic mice were labeled with CTV (Thermo Fisher Scientific) and cultured for 4 days in high-glucose DMEM (Thermo Fisher Scientific) supplemented with 10% fetal calf serum (Omega Scientific), 50 μM 2-mercaptoethanol (Thermo Fisher Scientific), 10 mM HEPES, nonessential amino acids (Thermo Fisher Scientific), and penicillin/streptomycin (Thermo Fisher Scientific). For islet-stimulated cultures, 25 islets were cultured with CTV-labeled splenotypes from NOD.8F10 mice for 4 days in 200 ml of complete DMEM in round-bottom 96-well plates. Islets were isolated from 6- to 10-week-old NOD.*Rag*^–/–^ mice by injecting 3 ml of ice-cold Cizyme into the common bile duct and digesting the pancreas at 37°C for 13 minutes. Digested islets were then washed with HBSS, resuspended in Lympholyte1.1 (Cedarlane), centrifuged at 800 *g* for 20 minutes at room temperature, washed with HBSS, and hand counted.

### CARs and retrovirus.

To generate the antigen-binding domain of the InsB-g7 CAR, the variable regions of the 1B2 mAb heavy and light chains were sequenced from hybridomas generated as previously described (31, 32). The DNA sequences were then converted into the scFv format and cloned into a murine stem cell virus–based (MSCV-based) retroviral vector where the scFvs were fused to the hinge (derived from mouse CD8), transmembrane (derived from mouse CD28), and intracellular CD28 and CD3ζ signaling domains. Retroviral particles were produced by using the Platinum-E (Plat-E) Retroviral Packaging Cell Line transfected with the pCL-Eco Retrovirus Packaging Vector, according to the manufacturer’s recommendations (Cell Biolabs). Control Tregs were either transduced with a Her2-CAR or left untransduced.

### Isolation, retroviral transduction, and expansion of CAR Tregs.

Spleens and LNs (popliteal, axillary, mandibular, and mesenteric) were harvested from 8- to 12-week-old NOD.*Foxp3^EGFP^* mice. The organs were dissociated to release single cells, and CD4^+^ cells were magnetically enriched using mouse negative selection CD4^+^ T cell isolation kits (STEMCELL Technologies and BioLegend). Live Tregs were sorted as fixable viability dye^neg^ (Thermo Fisher Scientific and Tonbo Biosciences), CD4^+^ (BD Biosciences and Tonbo Biosciences), CD25^+^ (BioLegend), and GFP^+^ using either a MoFlo Astrios (Beckman Coulter) or FACSAria II (BD Biosciences) cell sorter. CD25^neg^, GFP^neg^, CD4^+^ Tconvs were sorted in parallel. Tregs and Tconvs were cultured in ImmunoCult-XF T Cell Expansion Medium (STEMCELL Technologies) supplemented with 50 μmol/L of β-mercaptoethanol and 100 units/mL of penicillin/streptomycin (Thermo Fisher Scientific). Sorted Treg cultures also contained 1,000 U/mL of IL-2 (Proleukin) and 50 nmol/L of rapamycin (Sigma-Aldrich), whereas Tconv cultures contained 100 U/mL of IL-2. Tregs and Tconvs were stimulated with mouse T-Activator CD3/CD28 Dynabeads (Thermo Fisher Scientific) at a bead-to-cell ratio of 3:1 and 2:1, respectively.

For transduction, 2 or 3 days after stimulation (for Tconvs or Tregs, respectively), retrovirus, Lipofectamine 2000 (2 μg/mL, Thermo Fisher Scientific), and hexadimethrine bromide (Polybrene, 1.6 μg/mL, MilliporeSigma) were added and cells were centrifuged for 90 minutes at 805*g* at 32°C. (17). IL-2 and rapamycin (for Tregs) were replenished when cell cultures were split. On day 7, CD3/CD28 Dynabeads were magnetically removed and Tregs and Tconvs were rested for 2 days with decreased IL-2 (300 and 30 U/mL, respectively) before use for functional in vitro assays. Transduction efficiency was assessed on day 5 and/or 7 after cell activation by cell-surface staining using mouse anti-CD4, anti-Myc, and fixable viability dye eF780. Treg purity was assessed by staining with anti-FOXP3 and anti-HELIOS. Excess Tregs and Tconvs generated during expansion were cryopreserved on day 7 after sort following bead removal. All T cells were cryopreserved in 90% ImmunoCult base media and 10% dimethyl sulfoxide.

### T cell activation and proliferation assays.

On day 9 after stimulation, after 2 days of cell resting, CAR Tregs and Tconvs were collected, counted, and washed prior to processing in preparation for their respective assays. APCs from NOD spleens were obtained by depleting T cells using the Mouse CD90.2 Positive Selection Kit (STEMCELL Technologies), then pulsed with 15 μM of the indicated peptides and cocultured with Tregs/Tconvs at a 1:1 ratio. Treg cocultures were supplemented with 100 U/mL IL-2. After 24 hours, cells were stained with anti-CD4, anti-Myc, anti-CD69, anti–CTLA-4, anti-LAP, and FVD eF780, and the expression of CD69, LAP, and CTLA-4 was assessed by flow cytometry. For proliferation assays, APCs were irradiated by x-ray at 20 Gy**,** then pulsed with 10 μM peptide. Tregs and Tconvs were labeled with Cell Proliferation Dye CPD eF450 (Thermo Fisher Scientific) and cocultured at a 1:1 APC/T cell ratio. CAR Tregs also received supplemental 100 U/mL IL-2 one day into incubation. Cells were stained with anti-CD4, anti-Myc, and FVD eF780, and the proliferation of CAR Tregs, Tconvs, and responder T cells was assessed by dilution of their respective cell proliferation dye signal.

### Suppression assays.

To measure suppression of DCs, spleens from NOD mice were dissociated and incubated with Spleen Dissociation Media (STEMCELL Technologies); then DCs were isolated using the EasySep Mouse CD11c Positive Selection Kit II (STEMCELL Technologies). While adding the CD11c selection antibody cocktail to the cells, an additional CD11c-BV786 antibody (Invitrogen) was added at the same time to allow for flow cytometry analysis the following day. CD11c^+^ DCs were labeled with CPD eF450 and plated in 96-well U-bottom plates at either 20,000 or 50,000 per well and pulsed with either 10 μM insulin P8E or HEL peptide; 100,000 CAR or control Tregs were added to the DCs with IL-2 (100 U/mL). Cells were stained with anti-Myc, anti-CD86, anti-CD80, anti-CD11c, anti-CD4, and FVD eF780. Expression of CD80 and CD86 on DCs was measured by flow cytometry after 1 or 2 days.

To measure suppression of BDC2.5 T cells, serial dilutions of CPD eF670-labeled CAR or control Tregs were added to wells with 100,000 CD11c^+^ DCs pulsed with 10 nM p63 and 10 μM insulin P8E peptides. After 24 hours, CD4^+^ T cells from BDC2.5 mice were isolated using the EasySep Mouse CD4^+^ T Cell Isolation Kit, labeled with CPD eF450, and 50,000 were added to wells with Tregs and DCs. After an additional 3 days, BDC2.5 T cell proliferation was assessed by flow cytometry and supernatants were collected for analysis with the mouse Th1/Th2/Th17 Cytokine Kit (BD Biosciences) and FCAP Array Software, version 3.0.1 (Soft Flow).

### In vivo experiments.

On day 7 after stimulation (4 days after retroviral transduction), Dynabeads were removed and cells were washed and resuspended in HBSS. CD4^+^ T cells were isolated from NOD.BDC2.5 TCR mice by negative magnetic enrichment using biotinylated antibodies directed against TER119, CD8α, CD11b, CD16/32, NK1.1, Gr-1. Ly6G, and B220 (Tobo Biosciences) and MojoSort streptavidin microbeads (BioLegend). Fifty thousand CD4^+^ BDC2.5 T cells and either 150,000 or 450,000 CAR Tregs were transferred on the same day via consecutive intravenous injection into 6- to 10-week-old female NOD.*Rag1^–/–^* mice. Blood glucose was monitored daily starting 7 days after cell transfer and mice with glucose greater than 250 mg/dL (13.9mM) for 2 or more consecutive days were considered diabetic.

For prevention of spontaneous autoimmune diabetes, 8- to 10-week-old female NOD mice were intravenously injected with 2.5 to 3 million CD45.2^+^CD4^+^ InsB-g7 CAR Tregs. 1B2 CAR Tregs were more than 90% FOXP3^+^ and 70% to 90% CAR^+^, as indicated by InsBP8E tetramer staining. Littermate cohoused mice injected with HBSS were used as controls. Blood glucose was monitored once per week until 30 weeks of age, with glucose greater than 250 mg/dL (13.9mM) for 2 or more consecutive days considered diabetic.

### Tetramers and flow cytometry.

To evaluate CAR specificity, cells were stained with various peptide:IA^g7^ tetramers. Soluble peptide:IA^g7^ proteins were produced using S2 insect cells as previously described and made into tetramers by conjugating the soluble peptide:IA^g7^ molecules with BV421 (BioLegend), PE, or APC (Prozyme/Agilent) streptavidin at a 4.5:1 ratio (63). The tetramers used were InsBP8E:IA^g7^, InsBP8G:IA^g7^, p63:IA^g7^, and HEL_11–25_:IA^g7^.

For in vivo experiments, single-cell suspensions were obtained from spleen and LNs by mechanical disruption. Lymphocytes were isolated from pancreas by collagenase P and DNase digestion, followed by Percoll density gradient centrifugation at 800 *g* for 20 minutes. Single-cell suspensions were stained with InsBP8E:IA^g7^ tetramers, antibodies against CD4, CD8a, CD11c, CD11b, F4/80, CD90.1, CD90.2, CD45.1, CD45.2, and PD-1, and fixable live/dead dye for 30 minutes at 4°C in the presence of Fc block (2.4G2; Bio X Cell), fixed and permeabilized (Tonbo Biosciences), and stained for intracellular antigens FOXP3, HELIOS, IFN-γ, TNF-α, CTLA-4, and Ki67 overnight at 4°C in permeabilization buffer. A complete list of antibodies can be found in Supplemental Table 1. Flow cytometry was performed on LSRII or Fortessa cytometers (BD Biosciences) and analyzed using FlowJo software, version 10.8 (BD Biosciences).

### Epifluorescent microscopy.

Pancreata were harvested and frozen in OCT compound (Sakura Finetek) as previously described (64), cut into 4 groups of 8 sequential 7 μm thick sections separated by a depth of 150 μm using a Leica CM1860 UV cryostat (Leica Microsystems), and mounted as duplicates on Fisherbrand ProbeOn Plus glass slides (Thermo Fisher Scientific). Slides were fixed in cold acetone (L10407-AU; Thermo Fisher Scientific) for 10 minutes and stored at –20°C for no more than 3 months. For imaging, slides were first warmed to 25°C and sections were hydrated in PBS for 10 minutes. Sections were then blocked with 5% BSA (9048-46-8; Sigma-Aldrich) in the presence of 1.67 μg/mL Fc block (2.4G2; Bio X Cell) in PBS for 1 hour at 25°C, followed by permeabilization in 5% BSA in the presence of 0.05% Tween-20 (Thermo Fisher Scientific) for 15 minutes at 25°C. Sections were then stained with guinea pig anti-insulin (A0564; Dako) at 1:1,000 and anti-FOXP3 (Alexa Fluor 488, FJK-16s; Thermo Fisher Scientific) at 1:100 overnight at 4°C in 5% BSA in the presence of 0.05% sodium azide (BP922I-500; Thermo Fisher Scientific), 0.5% Triton X-100 (161-0407; Bio-Rad Laboratories), and 1.67 μg/mL FC block. Pancreas sections were then stained with secondary and direct conjugate antibodies for 1 hour at 25°C in 5% BSA. The secondary antibody used was donkey anti-guinea pig IgG (H and L chain) (Alexa Fluor 647, 706605148; Jackson ImmunoResearch) at 1:1,000. Fluorophore-conjugated antibodies used at 1:100 included anti-CD45.2 (BV421, 104; BioLegend), anti-CD4 (Alexa Fluor 594, GK1.5; BioLegend), anti-CD8a (PE, 53-6.7; BD Biosciences), and anti-Vβ4 (PE, KT4; BD Biosciences). Slides were mounted with ProLong Diamond Antifade Reagent (P36961; Thermo Fisher Scientific) using Gold Seal coverslips (12-518-108A; Thermo Fisher Scientific). Images were acquired on a Leica DM6000B epifluorescent microscope with a ×20 objective and quantified using a custom-built macro (65).

### Statistics.

The figures were generated using Adobe Illustrator, version 26, and Graph Pad Prism, version 9.4 (Graph Pad Software LLC). Data displayed in bar graphs are represented as means ± SEM. Statistics tests applied to the data are listed in the figure legends and were calculated using Graph Pad Prism. A *P* value of less than 0.05 was considered to be significant.

### Study approval.

All animal experiments were approved by the Institutional Animal Care and Use Committee of the University of Minnesota (2001-37804A) or the University of British Columbia Animal Care Committee (A20-0017).

### Data availability.

Values for all data points in graphs are reported in the Supporting Data Values file.

## Author contributions

JAS, VF, CMW, MM, PCO, CBV, BTF, and MKL designed the study. JAS, VF, CMW, MHA, YC, LAS, AJD, MEW, JSM, MM, PCO, and BTF performed the experiments. NS, MEW, and MHA provided animal husbandry. JAS, VF, CMW, MHA, AJD, MEW, JSM, BTF, and MKL analyzed the data. JAS prepared the figures. BTF and JAS prepared the graphical abstract. JAS, BTF, and MKL wrote the manuscript. All authors edited the manuscript. JAS and VF are co–first authors; the order reflects the relative contribution to the design and execution of the in vivo experiments. BTF and MKL are co–senior authors on the basis of the relative contribution to the initial conception and design of the study.

## Supplementary Material

Supplemental data

Supporting data values

## Version 1 08/10/2023

In-Press Preview

## Version 2 09/15/2023

Electronic publication
