# Supplementary material for: Tregs with an MHC class II peptide–specific chimeric antigen receptor prevent autoimmune diabetes in mice
Source: J Clin Invest. 2023 Sep 15;133(18):e168601. doi: 10.1172/JCI168601 (PMC10503798; doi:10.1172/JCI168601)
Supplement: Supplemental data [file jci-133-168601-s255.pdf]

A

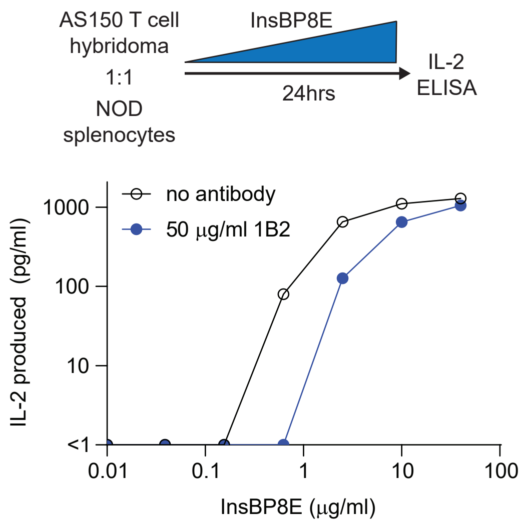

Supplemental figure 1. 1B2 antibody inhibits IL-2 release by InsBP8E stimulated AS150 T cell hybridoma. AS150 T cell hybridomas were cultured for 24hrs in the presence of various concentrations of InsBP8E peptide and IL-2 in the hybridoma supernatant was measure by ELISA. Data are representative of two independent experiments, n=1/group.

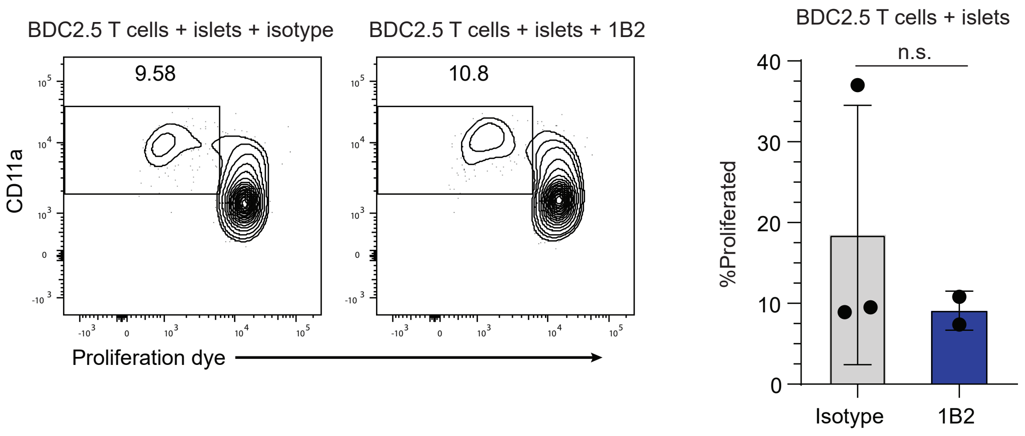

Supplemental figure 2. Flow cytometry plots (left) showing CD11a expression, as a marker of activation, and proliferation of BDC2.5 T cells after 3 days in culture with splenocytes as APCs and NOD islets, in the presence of either IgG1 isotype or 1B2 antibody. The panel of the right shows quantification of proliferation from plots shown on the left. Data is representative of 3 independent experiments, n=2-3/group. Students T-test, n.s.=not significant.

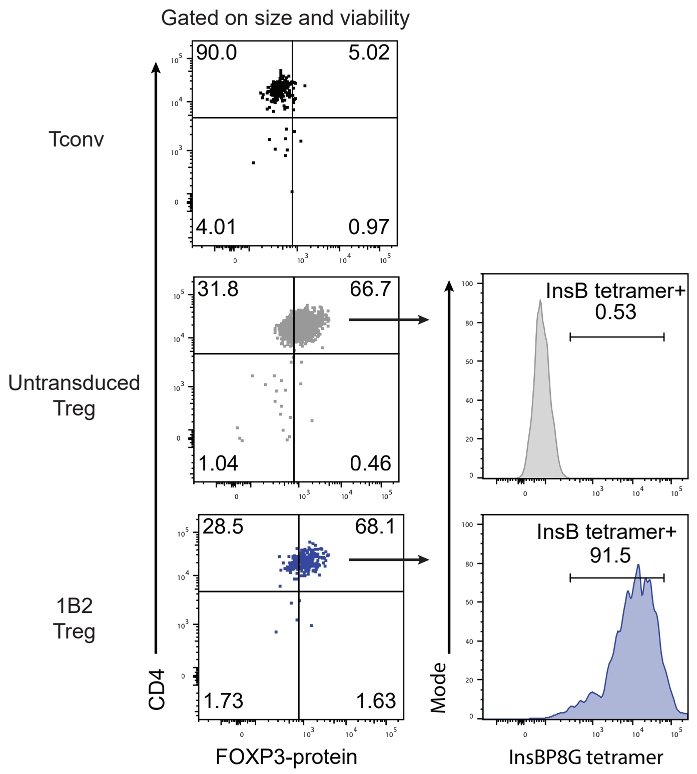

Supplemental figure 3. Representative flow cytometry plots of cells after 5 days in culture which were used for in vivo experiments. FOXP3 expression was used to determine Treg purity and InsBP8G tetramers were used to determine InsB-g7 CAR retrovirus transduction efficiency.

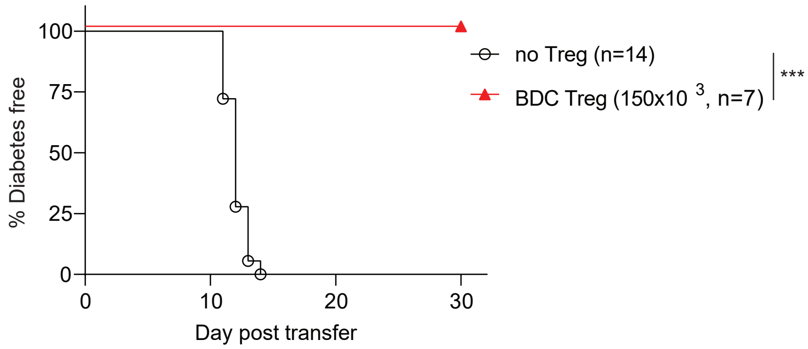

Supplemental figure 4. BDC2.5 Tregs suppress BDC 2.5 T cell-induced autoimmune diabetes. Survival curve for 6-10wk old NOD.*Rag*$^{-/-}$ mice recieving 150x10^3(3:1) in vitro expanded BDC2.5 Tregs cotransferred with 50x10^3 naïve BDC2.5 CD4$^+$ T cells. Data is from 1 experiment, no Treg n=14, 150x10^3 BDC2.5 Treg n=7. Log-rank test with Bonferroni correction, ***P<0.001.

Supplemental Table 1. Antibodies used for flow cytometry

Antigen	Fluor	Clone	Supplier	Catalogue number
CD4	PE	RM4-5	Tonbo Biosciences	50-0042
		GK1.5	BD Biosciences	557308
	BUV496	GK1.5	BD Biosciences	612952
	BV605	RM4-5	BD Biosciences	563151
CD11b	APC-ef780	M1170	ThermoFisher	47-0112-82
CD11c	APC-ef780	N418	ThermoFisher	47-0114-82
	SB780	N418	ThermoFisher	78-0114-80
B220	APC-ef780	RA3-6B2	ThermoFisher	47-0452-82
Thy1.1	BUV737	OX7	BD Biosciences	612837
Thy1.2	BV786	30-H12	BioLegend	105331
CD45.1	BV711	A20	BioLegend	110739
CD45.2	BUV395			
PD-1	PerCp-ef710	J43	ThermoFisher	46-9985-82
Fixable L/D dye	Ghost dye Red-780		Tonbo Biosciences	13-0865
Fixable Viability Dye eFluor™ 780	eF780		ThermoFisher	65-0865-18
Cell Proliferation Dye eFluor™ 450	eF450		ThermoFisher	65-0842-90
Cell Proliferation Dye eFluor™ 670	eF670		ThermoFisher	65-0840-90
Foxp3	AF488	FJK-16s	ThermoFisher	53-5773-82
	PE	FJK-16s	ThermoFisher	12-5773-82
Ki67	BUV737	solA15	ThermoFisher	367-5698-82
	BV421	16A8	BioLegend	652411
CTLA4	PE	4C10-4B9	BioLegend	563151
Helios	PE-Cy7	22F6	BioLegend	137236
	eF450	22F6	ThermoFisher	48-9883-42
IFNγ	BV650	XMG1.2	BioLegend	505532
TNFα	Ef450	mAb11	ThermoFisher	48-7349-42
c-Myc	AF647	9E10	UBC Ablab	67-0029-01
LAP	PE	TW7-16B4	ThermoFisher	12-9821-82
CD69	BV785	H1.2F3	Biolegend	104543
CD152 (CTLA-4)	BV605	UC10-4B9	Biolegend	106323
CD86	BV650	GL1	BD Biosciences	564200
CD80	BUV395	16-10A1	BD Biosciences	740246
